# A population-based study on the burden of hospitalized pediatric pneumococcal disease in Taiwan before and after the introduction of 13-valent pneumococcal conjugate vaccine into the childhood immunization program in 2015

**DOI:** 10.1186/s12879-024-10379-z

**Published:** 2025-02-05

**Authors:** Ting-An Yen, Jhong-Lin Wu, Chi-Chuan Wang, Ling-Ya Huang, See-Hwee Yeo, Dony Patel, Cindy Thiow Koon Lim, Hung-Wei Lin, Eriko Yamada, Isaya Sukarom

**Affiliations:** 1 Department of Pediatrics, National Taiwan University Hospital, Taipei, Taiwan; 2 Department of Emergency Medicine, National Taiwan University Hospital, Taipei, Taiwan; 3 School of Pharmacy, College of Medicine, National Taiwan University, Taipei, Taiwan; 4 Graduate Institute of Clinical Pharmacy, College of Medicine, National Taiwan University Hospital, Taipei, Taiwan; 5 Department of Pharmacy, National Taiwan University Hospital, Taipei, Taiwan; 6 Real World Solutions, IQVIA Solutions Asia, Singapore, Singapore; 7 Global Database Studies, Real World Solutions, IQVIA, London, UK; 8 Real World Solutions, IQVIA Solutions Taiwan, Taipei, Taiwan; 9 Global Medical and Scientific Affairs, MSD Singapore, Singapore, Singapore; 10 Regional Outcomes Research, MSD Thailand, Bangkok, Thailand; 11 999/9 The Offices at Central World, Rama I Road, Bangkok, Thailand; 12 9 Battery Road, #17-01 MYP Centre, Singapore, 049910 Singapore

**Keywords:** Pneumococcal conjugate vaccine, Invasive pneumococcal disease, Non-bacteremic pneumococcal pneumonia, Acute otitis media, Taiwan

## Abstract

**Background:**

To estimate the burden of invasive pneumococcal disease, non-bacteremic pneumococcal pneumonia, and acute otitis media before and after inclusion of the 13-valent pneumococcal vaccine (PCV13) into Taiwan’s Childhood Immunization Program in 2015.

**Methods:**

Episodes of eligible children aged < 18 years hospitalized with invasive pneumococcal disease, non-bacteremic pneumococcal pneumonia, or acute otitis media between 1 January 2011 and 31 December 2019 were identified from the National Health Insurance Research Database. Annual hospitalized incidence rate, case fatality rate, and healthcare resource utilization and costs were estimated. Incidence time trends were assessed with interrupted time series analyses.

**Results:**

1,284 invasive pneumococcal disease episodes, 25,074 non-bacteremic pneumococcal pneumonia episodes, and 23,139 acute otitis media episodes were identified. The overall annual incidence rates of invasive pneumococcal disease, non-bacteremic pneumococcal pneumonia, and acute otitis media were 3.31, 64.61, and 59.62 episodes per 100,000 person-years, respectively. Interrupted time series analyses results showed a significantly lower baseline incidence rate (incidence rate ratio [IRR]:0.58, p-value = 0.001) for invasive pneumococcal disease, and significantly higher baseline incidence rate (IRR:1.17, p-value < 0.001) for non-bacteremic pneumococcal pneumonia in the post-PCV13 period. Baseline incidence rates between the two periods were comparable for acute otitis media. A significant increase in trend of incidence rate was observed for all three diseases. Case fatality rate was 1.79%, 0.09%, and 0.00% for invasive pneumococcal disease, non-bacteremic pneumococcal pneumonia, and acute otitis media, respectively. Median length of hospitalization per inpatient visit was comparable between the two periods for invasive pneumococcal disease and non-bacteremic pneumococcal pneumonia, but significantly shorter in the post-PCV13 period for acute otitis media. In the post-PCV13 period, average total costs per episode was lower for invasive pneumococcal disease and non-bacteremic pneumococcal pneumonia, but higher for acute otitis media.

**Conclusions:**

Residual clinical and economic burden of pneumococcal diseases remained substantial after PCV13 inclusion into Taiwan’s Childhood Immunization Program. To further reduce the disease burden among children, additional research to investigate the cause of increasing trends of hospitalized invasive pneumococcal disease, non-bacteremic pneumococcal pneumonia and acute otitis media in the post-PCV13 era will be required.

**Supplementary Information:**

The online version contains supplementary material available at 10.1186/s12879-024-10379-z.

## Background

*Streptococcus pneumoniae (S. pneumoniae*) is a major cause of morbidity and mortality in children, causing a broad clinical spectrum of infections ranging from mild illness such as acute otitis media (AOM) to more serious invasive diseases such as meningitis and bacteremic pneumococcal pneumonia. In 2015, an estimated 3.7 million episodes of serious pneumococcal diseases and approximately 294,000 deaths occurred globally in children < 5 years [1].

Pneumococcal conjugate vaccines (PCVs) have been developed to prevent infections of *S. pneumoniae*. PCV is an inactivated vaccine containing polysaccharides from serotypes of *S. pneumoniae* which most commonly cause invasive disease. PCVs that include 7 (PCV7), 10 (PCV10), and 13 (PCV13) serotypes have been introduced into the national childhood immunization program (CIP) in many countries, and shown to decrease the risk of invasive pneumococcal disease (IPD) and pneumonia, especially in very young children [2–13]. However, studies assessing the impact of PCV on AOM incidence have reported mixed results [14–19].

In Taiwan, PCV7 was first introduced for use in the private sectors in 2006. In 2009, it was provided free-of-charge for selected high-risk children aged ≤ 5 years, and also for children from low-income households in 2010 [20]. PCV7 was replaced by PCV10 in 2010, and PCV13 subsequently replaced PCV10 in 2013 [20–22]. PCV13 was used in the national catch-up program for children aged 1–5 years between 2013 and 2014, and later included into the CIP in 2015 [23]. While studies in Taiwan have shown the effectiveness of PCV13 in reducing IPD and pneumonia incidence following the national catch-up program, the impact of PCV13 on AOM has yet to be evaluated [20, 24, 25]. In addition, it would be of interest to assess the burden of pneumococcal diseases after inclusion of PCV13 into Taiwan’s CIP.

Thus, this study aims to quantify the clinical and economic burden of pneumococcal diseases by examining the hospitalized incidence rate (IR), case fatality rate (CFR), healthcare resource utilization (HCRU) and costs associated with IPD, non-bacteremic pneumococcal pneumonia, and AOM in children, before and following the introduction of PCV13 into Taiwan’s CIP.

## Methods

### Study design and study population

A retrospective observational study was conducted using the National Health Insurance Research Database (NHIRD), which covers approximately 99% of Taiwan’s population [26]. Data were extracted from children aged < 18 years hospitalized with IPD, non-bacteremic pneumococcal pneumonia, or AOM, between 1 January 2011 and 31 December 2019. The International Classification of Diseases (ICD), Ninth and Tenth Revisions, were used to identify pneumococcal-specific disease episodes during the study period (ICD codes are provided in Additional File 1). Otitis media with effusion was considered as AOM, since both conditions are clinically challenging to differentiate.

The date of first hospitalization marked the start of a disease episode. The date of hospital discharge marked the end of a disease episode if there were no other hospitalizations with the same diagnostic codes occurring within a pre-specified time period from date of discharge. Any subsequent hospitalization with the same diagnostic codes that occurred within the pre-specified time period was assumed to be related to the first episode and collapsed to form a single episode. A time period of  90 days was used for IPD and non-bacteremic pneumococcal pneumonia hospitalizations, while 14 and 28 days were used for AOM and otitis media with effusion hospitalizations respectively [27–29].

### Statistical analyses

Annual hospitalized IRs for each disease, defined as the number of new disease episodes in a calendar year, were estimated for each year during the study period. Interrupted time series analyses (ITSAs) were conducted on quarterly IR time trends to examine the impact of PCV13 introduction into Taiwan’s CIP. A Poisson or negative binomial regression model was fitted (as appropriate) to observed quarterly IRs in the pre-PCV13 (2011–2014) and post-PCV13 (2016–2019) periods. Incidence rate ratios (IRRs) were computed to compare the baseline and trends of IRs between the two time periods. Indicator variables for each quarter of the year were also included to adjust for any seasonality. The year 2015 was considered as a transition period and excluded from analyses since it was the year of PCV13 inclusion into the CIP.

CFRs for each disease, defined as the proportion of deaths among disease episodes, were estimated for pre-PCV13 and post-PCV13 periods.

HCRU and costs associated with disease episodes were summarized using descriptive statistics, and compared between pre-PCV13 and post-PCV13 periods using Student’s t-test or Kruskal-Wallis test. HCRU included length of hospitalization as a key component. Cost components (in New Taiwan Dollars, NTD) included inpatient, outpatient and emergency department visits, as well as outpatient pharmacy costs. HCRU and costs were reported for disease episodes identified up till 31 December 2018, to allow time for follow-up and discharge from hospital. Costs were analysed from the healthcare system perspective, and no adjustment was applied to costs across years since reimbursement for health services and medications have remained stable under the National Health Insurance system [30].

Sensitivity analyses were conducted to evaluate the impact of using pneumococcal-specific and non-specific ICD codes (compared to pneumococcal-specific ICD codes) on the estimation of annual hospitalized IRs and ITSA results. These were only conducted for IPD and non-bacteremic pneumococcal pneumonia, since there were no non-specific ICD codes identified for AOM.

Separate analyses were conducted for IPD, non-bacteremic pneumococcal pneumonia and AOM. For each disease, analyses were performed for all children < 18 years, and for specific age groups of < 5 years and 5 to < 18 years. All analyses were performed using SAS version 9.2 (SAS Institute Inc., North Carolina, United States of America). A *P*-value < 0.05 was considered statistically significant.

## Results

A total of 49,497 hospital episodes among children were identified between 2011 and 2019, of which 1,284 were IPD episodes, 25,074 were non-bacteremic pneumococcal pneumonia episodes, and 23,139 were AOM episodes.

### IPD

**Fig. 1:**
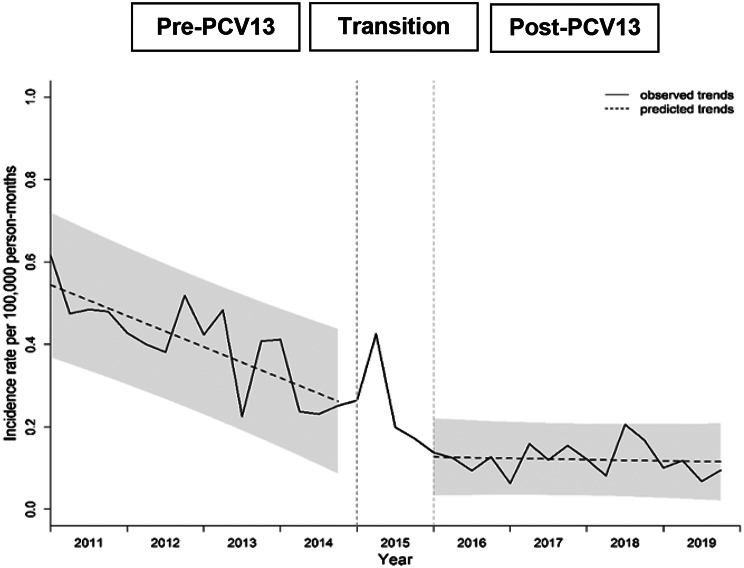
Trend in quarterly incidence of hospitalized invasive pneumococcal disease in children from 2011–2019

Annual hospitalized IRs for IPD among all children ranged between 1.16 and 6.26 episodes per 100,000 person-years, with an overall IR of 3.31 episodes per 100,000 person-years during the study period. Overall IPD IR was 7.8 times higher in children aged < 5 years, compared with those 5 to < 18 years (9.27 vs. 1.19 episodes per 100,000 person-years). ITSA results for quarterly IPD IRs in children < 18 years showed a significantly lower baseline IR and a significant increase in the trend of IR in the post-PCV13 period, compared with pre-PCV13 period (Fig. 1; Table 1). ITSA was not performed by age groups due to low counts.

**Table 1:** IRRs of baseline IR and trend of IR in post-PCV13 period compared to pre-PCV13 period

	Baseline IR		Trend of IR	
Condition	IRR	*P*-value	IRR	*P*-value
**Invasive pneumococcal disease**				
< 18 years	0.58	0.001*	1.04	0.010*
**Non-bacteremic pneumococcal pneumonia**				
< 18 years	1.17	< 0.001*	1.07	< 0.001*
< 5 years	1.14	0.003*	1.07	< 0.001*
5 to < 18 years	1.35	< 0.001*	1.17	< 0.001*
**Acute otitis media**				
< 18 years	1.01	0.779	1.01	0.036*
< 5 years	0.97	0.502	1.01	0.010*
5 to < 18 years	1.06	0.318	1.05	< 0.001*

Abbreviations: IR, incidence rate; IRR, incidence rate ratio; PCV, pneumococcal conjugate vaccine

Overall CFR for IPD was higher in children aged 5 to < 18 years (2.05%), compared with children < 5 years (1.70%) (Table 2). For all age groups, CFR was lower in the post-PCV13 period than pre-PCV13 period.

**Table 2:** Case fatality rates from 2011–2019, by pneumococcal disease, age groups and PCV13 periods

	Overall			Pre-PCV13			Post-PCV13		
Condition	Cases (*n*)	Deaths (*n*)	CFR (%)	Cases (*n*)	Deaths (*n*)	CFR (%)	Cases (*n*)	Deaths (*n*)	CFR (%)
**Invasive pneumococcal disease**									
< 18 years	1,284	23	1.79	906	19	2.10	239	1	0.42
< 5 years	943	16	1.70	668	14	2.10	172	0	0.00
5 to < 18 years	341	7	2.05	238	5	2.10	67	1	1.49
**Non-bacteremic pneumococcal pneumonia**									
< 18 years	25,074	22	0.09	10,962	15	0.14	11.947	4	0.03
< 5 years	18,368	15	0.08	7,751	11	0.14	9,118	2	0.02
5 to < 18 years	6,706	7	0.10	3,211	4	0.12	2,829	2	0.07
**Acute otitis media**									
< 18 years	23,139	0	0.00	12,256	0	0.00	8,700	0	0.00
< 5 years	14,948	0	0.00	7,884	0	0.00	5,623	0	0.00
5 to < 18 years	8,191	0	0.00	4,372	0	0.00	3,077	0	0.00

Abbreviations: CFR, case fatality rate; PCV, pneumococcal conjugate vaccine

Median length of hospitalization per inpatient visit in children < 18 years was comparable between pre-PCV13 and post-PCV13 periods (7 days) (Table 3). Median costs per visit for inpatient and outpatient visits in children < 18 years were significantly higher (both p-values < 0.001) in the post-PCV13 period, compared with pre-PCV13 period. Similar inferences were made for age groups < 5 years and 5 to < 18 years. However, the average total costs per episode in children < 18 years and < 5 years was 9.0% and 15.8% lower in the post-PCV13 period than pre-PCV13 period, respectively.

**Table 3:** IPD-associated HCRU and costs during pre-PCV13 and post-PCV13 periods, by age groups

	< 18 years			< 5 years			5 to < 18 years		
	Pre-PCV13 (*n* = 906)	Post-PCV13 (*n* = 194)	*P*-value	Pre-PCV13 (*n* = 668)	Post-PCV13 (*n* = 142)	*P*-value	Pre-PCV13 (*n* = 238)	Post-PCV13 (*n* = 52)	*P*-value
**Healthcare resource utilization**									
Inpatient									
Number of visits ^a^, n	922	200	-	681	144	-	241	56	-
Length of hospitalization per visit (days)									
Mean ± SD	9.89 ± 9.89	9.38 ± 7.12	0.497	9.91 ± 9.38	9.03 ± 6.99	0.201	9.83 ± 11.22	10.35 ± 7.47	0.683
Median (Q1, Q3)	7 (5, 11)	7 (5, 11)	0.342	7 (5, 11)	7 (5, 10)	0.451	7 (5, 11)	7 (5, 14)	0.156
Total length of hospitalization ^b^	8,960	1,820	-	6,621	1,282	-	2,339	538	-
Per episode									
Average number of visits	1.02	1.03	-	1.02	1.01	-	1.01	1.08	-
Average length of hospitalization (days)	9.89	9.38	-	9.91	9.03	-	9.83	10.35	-
Outpatient									
Number of visits ^c^, n	324	73	-	260	44	-	64	29	-
Average number of visits per episode	0.36	0.38	-	0.39	0.31	-	0.27	0.56	-
ED									
Number of visits ^d^, n	38	5	-	25	NR	-	13	NR	-
Average number of visits per episode	0.04	0.03	-	0.04	ND	-	0.06	ND	-
**Costs**									
Inpatient									
Per visit									
Mean ± SD	70,215.72 ± 157,924.88	63,811.78 ± 85,838.24	0.584	70,068.68 ± 146,431.21	59,114.33 ± 87,095.60	0.390	70,628.41 ± 186,789.51	76,639.42 ± 81,745.38	0.820
Median (Q1, Q3)	22,983.50 (15,082.00, 43,331.00)	30,967.50 (20,350.00, 60,114.00)	< 0.001*	22,854.00 (15,354.50, 42,235.00)	30,091.50 (20,350.00, 44,104.00)	< 0.001*	23,463.00 (13,515.00, 46,935.00)	33,366.50 (20,077.00, 102,065.00)	0.001*
Total inpatient costs	63,615,439	12,379,485	-	46,805,877	8,394,235	-	16,809,562	3,985,250	-
Average inpatient costs per episode	70,215.72	63,811.78	-	70,068.68	59,114.33	-	70,628.41	76,639.42	-
Outpatient									
Per visit (excluding pharmacy costs)									
Mean ± SD	1,084.92 ± 2,472.11	2,026.84 ± 2,744.84	0.036*	1,126.99 ± 2,577.35	1,581.13 ± 1,776.43	0.277	926.70 ± 2,045.39	2,849.69 ± 3,927.24	0.111
Median (Q1, Q3)	373.00 (323.00, 885.00)	981.00 (422.00, 2,128.00)	< 0.001*	373.00 (362.00, 885.00)	834.50 (422.00, 1,995.00)	0.005*	397.50 (314.00, 878.00)	1,032.00 (559.00, 3,240.00)	0.002*
Total outpatient visit costs	237,597	74,993	-	194,969	37,947	-	42,628	37,046	-
Pharmacy costs per visit									
Mean ± SD	327.76 ± 1,072.40	272.52 ± 270.75	0.799	369.37 ± 1,201.63	211.64 ± 186.62	0.626	177.73 ± 269.61	350.00 ± 344.77	0.095
Median (Q1, Q3)	99.50 (52.50, 208.50)	167.00 (77.00, 370.00)	0.033*	99.00 (43.00, 217.00)	169.50 (52.00, 304.00)	0.150	103.00 (69.00, 146.00)	167.00 (81.00, 691.00)	0.055
Total outpatient pharmacy costs	49,820	6,813	-	43,955	2,963	-	5,865	3,850	-
Total outpatient costs ^e^	287,417	81,806	-	238,924	40,910	-	48,493	40,896	-
Per episode									
Average outpatient visit costs	262.25	386.56	-	291.87	267.23	-	179.11	712.42	-
Average pharmacy costs	54.99	35.12	-	65.80	20.87	-	24.64	74.04	-
Average outpatient costs	317.24	421.68	-	357.67	288.10	-	203.75	786.46	-
ED									
Per visit									
Mean ± SD	4,418.92 ± 4,367.28	4,468.20 ± 1,006.31	0.954	4,892.20 ± 5,081.51	ND	-	3,508.77 ± 2,408.61	ND	-
Median (Q1, Q3)	3,180.50 (2,210.00, 4,501.00)	4,385.00 (4,012.00, 5,212.00)	0.083	3,377.00 (2,311.00, 4,501.00)	ND	-	2,834.00 (1,846.00, 3,330.00)	ND	-
Total ED costs	167,919	22,341	-	122,305	10,855	-	45,614	11,486	-
Average ED costs per episode	185.34	115.16	-	183.09	76.44	-	191.66	220.89	-
Total costs ^f^	64,070,775	12,483,632	-	47,167,106	8,446,000	-	16,903,669	4,037,632	-
Average total costs per episode	70,718.30	64,348.62	-	70,609.44	59,478.87	-	71,023.82	77,646.77	-

Abbreviations: ED, emergency department; HCRU, healthcare resource utilization; IPD, invasive pneumococcal disease; ND, not determined; NR, not reported (counts < 3); PCV, pneumococcal conjugate vaccine; Q1, 25th percentile; Q3, 75th percentile; SD, standard deviation

^a^ Number of inpatient visits is defined as the number of IPD-associated hospital admissions

^b^ Total inpatient hospitalization is defined as the total duration of stay (in days) for all IPD-associated hospitalizations

^c^ Number of outpatient visits is defined as the number of hospital outpatient visits that occur on separate dates. Multiple records on the same date will be considered as a single visit

^d^ Number of ED visits is defined as the number of IPD-associated visits to the hospital ED that occur on separate dates

^e^ Total outpatient costs is defined by the sum of total outpatient visit costs and total outpatient pharmacy costs

^f^ Total costs is defined by the sum of total inpatient costs, total outpatient costs and total ED costs

## Non-bacteremic pneumococcal pneumonia

**Fig. 2:**
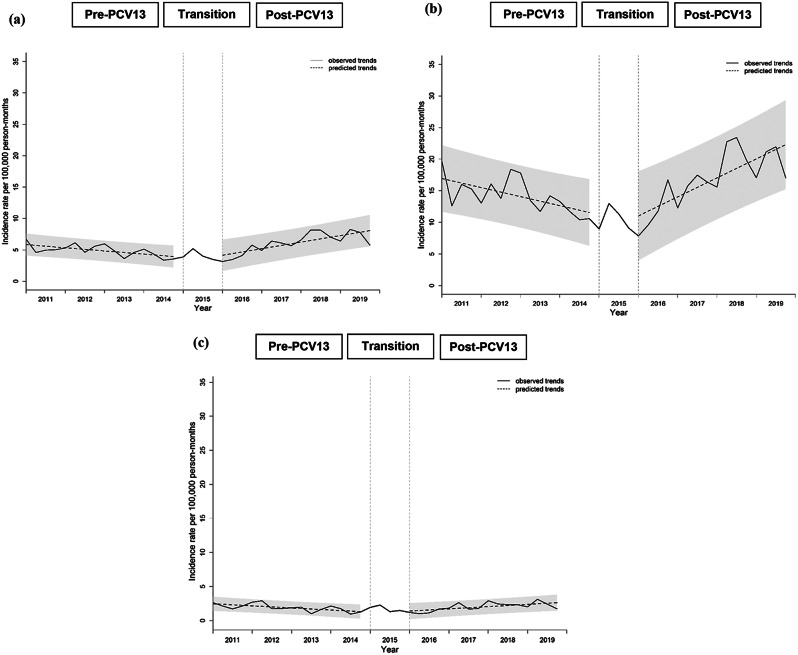
Trend in quarterly incidence of hospitalized non-bacteremic pneumococcal pneumonia in children from 2011-2019. **(a)** Children <18 years; **(b)** Children <5 years; **(c)** Children 5 to <18 years

Annual hospitalized IRs for non-bacteremic pneumococcal pneumonia among all children ranged between 49.86 and 91.09 episodes per 100,000 person-years, with an overall IR of 64.61 episodes per 100,000 person-years during the study period. Overall non-bacteremic pneumococcal pneumonia IR was 7.7 times higher in children aged < 5 years, compared with those 5 to < 18 years (180.53 vs. 23.42 episodes per 100,000 person-years). ITSA results for quarterly non-bacteremic pneumococcal pneumonia IRs in children < 18 years showed a significantly higher baseline IR and a significant increase in trend of IR in the post-PCV13 period, compared with pre-PCV13 period (Fig. 2; Table 1). These were similarly observed in age groups < 5 years and 5 to < 18 years.

Overall CFR for non-bacteremic pneumococcal pneumonia was higher in children aged 5 to < 18 years (0.10%), compared with children < 5 years (0.08%) (Table 2). For all age groups, CFR was lower in the post-PCV13 period than pre-PCV13 period.

Median length of hospitalization per inpatient visit in children < 18 years was comparable between pre-PCV13 and post-PCV13 periods (5 days) (Table 4). Median costs per visit for inpatient and ED visits in children < 18 years were significantly higher in the post-PCV13 period, compared with pre-PCV13 period (both p-values < 0.001). These were similarly observed in age groups < 5 years and 5 to < 18 years. Median outpatient visit costs in the post-PCV13 period was significantly lower for children < 18 years and 5 to < 18 years, but significantly higher for those < 5 years (all p-values < 0.001), when compared with pre-PCV13 period. Average total costs per non-bacteremic pneumococcal pneumonia episode was approximately 1.0-4.3% lower in the post-PCV13 period than pre-PCV13 period across all age groups.

**Table 4:** Non-bacteremic pneumococcal pneumonia-associated HCRU and costs during pre-PCV13 and post-PCV13 periods, by age groups

	< 18 years			< 5 years			5 to < 18 years		
	Pre-PCV13 (*n* = 10,962)	Post-PCV13 (*n* = 8,624)	*P*-value	Pre-PCV13 (*n* = 7,751)	Post-PCV13 (*n* = 6,581)	*P*-value	Pre-PCV13 (*n* = 3,211)	Post-PCV13 (*n* = 2,043)	*P*-value
**Healthcare resource utilization**									
Inpatient									
Number of visits ^a^, n	11,571	9,693	-	1,617	7,441	-	3,313	2,252	-
Length of hospitalization per visit (days)									
Mean ± SD	5.82 ± 5.06	5.69 ± 4.40	0.063	5.37 ± 3.27	5.68 ± 4.33	0.034*	5.76 ± 5.33	5.73 ± 4.63	0.859
Median (Q1, Q3)	5 (4, 6)	5 (4, 6)	0.139	5 (3, 6)	5 (4, 6)	0.115	5 (3, 6)	5 (3, 6)	0.396
Total length of hospitalization ^b^	63,810	49,099	-	45,314	37,383	-	18,496	11,716	-
Per episode									
Average number of visits	1.06	1.12	-	1.07	1.13	-	1.03	1.10	-
Average length of hospitalization (days)	5.82	5.69	-	5.85	5.68	-	5.76	5.74	-
Outpatient									
Number of visits ^c^, n	12,839	8,854	-	9,245	7,315	-	3,594	1,539	-
Average number of visits per episode	1.17	1.03	-	1.19	1.11	-	1.12	0.75	-
ED									
Number of visits ^d^, n	2,404	1,188	-	1,718	964	-	686	224	-
Average number of visits per episode	0.22	0.14	-	0.22	0.15	-	0.21	0.11	-
**Costs**									
Inpatient									
Per visit									
Mean ± SD	24,256.66 ± 68,933.02	23,736.67 ± 40,639.74	0.511	24,950.92 ± 69,804.31	24045.96 ± 43346.04	0.344	22,580.80 ± 66763.93	22,740.40 ± 30,309.87	0.919
Median (Q1, Q3)	14,408.00 (10,794.00, 20,469.00)	17,948.50 (13,565.50, 24,395.50)	< 0.001*	14,835.00 (11,176.00, 20,900.00)	18,241 (13,903.00, 24,703.00)	< 0.001*	13,423.00 (9,941.00, 19,374.00)	16,985.00 (12,600.00, 23,546.00)	< 0.001*
Total inpatient costs	265,901,510	204,705,071	-	193,394,576	158,246,436	-	72,506,934	46,458,635	-
Average inpatient costs per episode	24,256.66	23,736.67	-	24,950.92	24045.96	-	22,580.80	22,740.40	-
Outpatient									
Per visit (excluding pharmacy costs)									
Mean ± SD	752.57 ± 768.09	855.60 ± 870.85	< 0.001*	780.18 ± 778.73	898.68 ± 913.68	< 0.001*	686.70 ± 738.09	681.40 ± 641.83	0.844
Median (Q1, Q3)	480 (357, 877)	444 (422, 1,012)	< 0.001*	431 (368, 924)	480 (422, 1084)	< 0.001*	507 (314, 803)	370 (359, 740)	< 0.001*
Total outpatient visit costs	5,782,031	4,285,706	-	4,223,912	3,609,080	-	1,558,119	676,626	-
Pharmacy costs per visit									
Mean ± SD	245.49 ± 1,465.14	201.17 ± 416.41	0.019*	259.71 ± 1,735.70	192.86 ± 278.17	0.009*	212.01 ± 325.70	234.68 ± 748.36	0.258
Median (Q1, Q3)	118.00 (54.00, 248.00)	104.00 (50.00, 231.00)	< 0.001*	117.00 (51.00, 253.00)	102.00 (48.00, 226.00)	< 0.001*	119.00 (60.00, 243.00)	116.00 (58.50, 241.00)	0.434
Total outpatient pharmacy costs	1,652,130	875,278	-	1,226,847	672,515	-	425,283	202,763	-
Total outpatient costs ^e^	7,434,161	5,160,984	-	5,450,759	4,281,595	-	1,983,402	879,389	-
Per episode									
Average outpatient visit costs	527.46	496.95	-	544.95	548.41	-	485.24	331.19	-
Average pharmacy costs	150.71	101.49	-	158.28	102.19	-	132.45	99.25	-
Average outpatient costs	678.18	598.44	-	703.23	650.60	-	617.69	430.44	-
ED									
Per visit									
Mean ± SD	3,043.67 ± 1,659.49	3,504.01 ± 1,913.48	< 0.001*	3,152.17 ± 1,755.76	3,539.51 ± 1,853.63	< 0.001*	2,777.96 ± 1,361.12	3,408.04 ± 1,951.53	< 0.001*
Median (Q1, Q3)	2,961.00 (2,161.00, 3,683.00)	3,469.00 (2,160.00, 4,434.00)	< 0.001*	3,072.00 (2,193.00, 3,787.00)	3,537.00 (2,254.00, 4,480.00)	< 0.001*	2,705.00 (2,127.00, 3,335.00)	3,249.00 (1,999.00, 4,268.50)	< 0.001*
Total ED costs	6,875,650	3,949,018	-	5,056,087	3,224,497	-	1,819,563	724,521	-
Average ED costs per episode	627.23	457.91	-	652.31	489.97	-	566.67	354.64	-
Total costs ^f^	280,211,321	213,815,073	-	203,901,422	165,752,528	-	76,309,899	48,062,545	-
Average total costs per episode	25,562.06	24,793.03	-	26,306.47	25,186.53	-	23,765.15	23,525.48	-

Abbreviations: ED, emergency department; HCRU, healthcare resource utilization; PCV, pneumococcal conjugate vaccine; Q1, 25th percentile; Q3, 75th percentile; SD, standard deviation

^a^ Number of inpatient visits is defined as the number of non-bacteremic pneumococcal pneumonia-associated hospital admissions

^b^ Total inpatient hospitalization is defined as the total duration of stay (in days) for all non-bacteremic pneumococcal pneumonia-associated hospitalizations

^c^ Number of outpatient visits is defined as the number of hospital outpatient visits that occur on separate dates. Multiple records on the same date will be considered as a single visit

^d^ Number of ED visits is defined as the number of non-bacteremic pneumococcal pneumonia-associated visits to the hospital ED that occur on separate dates

^e^ Total outpatient costs is defined by the sum of total outpatient visit costs and total outpatient pharmacy costs

^f^ Total costs is defined by the sum of total inpatient costs, total outpatient costs and total ED costs

### AOM

**Fig. 3:**
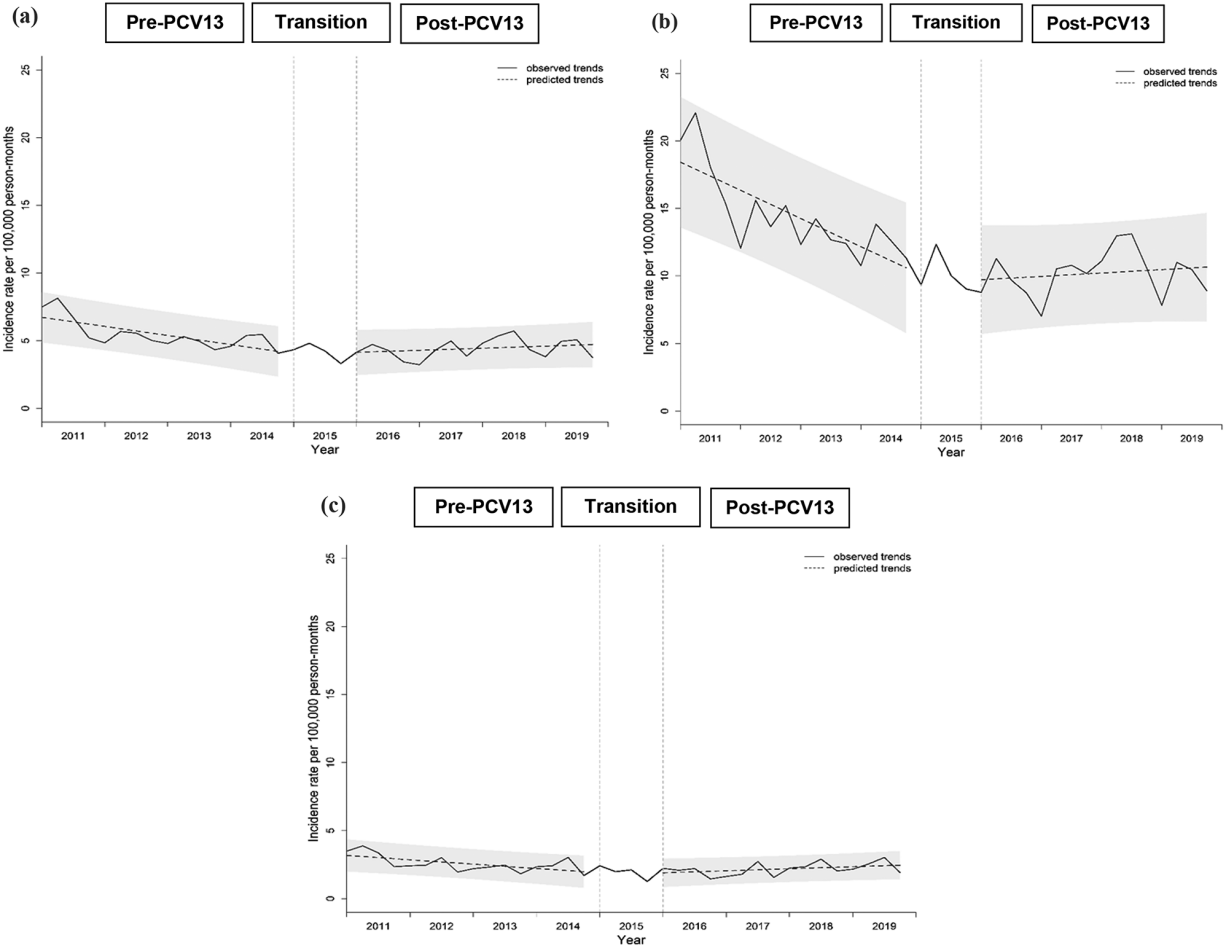
Trend in quarterly incidence of hospitalized acute otitis media in children from 2011-2019. **(a)** Children <18 years; **(b)** Children <5 years; **(c)** Children 5 to <18 years

Annual hospitalized IRs for AOM among all children ranged between 49.79 and 83.86 episodes per 100,000 person-years, with an overall IR of 59.62 episodes per 100,000 person-years during the study period. Overall AOM IR was 5.1 times higher in children aged < 5 years, compared with those 5 to < 18 years (146.92 vs. 28.61 episodes per 100,000 person-years). While ITSA results for quarterly AOM IRs among children < 18 years did not show a significant change in baseline IR, a marginally significant increase in trend of IR was observed in the post-PCV13 period, compared with pre-PCV13 period (Fig. 3; Table 1). These were similarly observed in age groups < 5 years and 5 to < 18 years.

Over the study period, there was no documented death due to AOM.

Median length of hospitalization per inpatient visit in children < 18 years was significantly shorter (p-value < 0.001) in the post-PCV13 period (3 days), compared with pre-PCV13 period (4 days) (Table 5). Median costs per visit in children < 18 years was significantly higher for inpatient, outpatient, and ED visits, but significantly lower for outpatient pharmacy costs in the post-PCV13 period, compared with pre-PCV13 period (all p-values < 0.001). These were similarly observed in children < 5 years and 5 to < 18 years. The average total costs per AOM episode was 18.0-19.4% higher in the post-PCV13 period than pre-PCV13 period for all age groups.

**Table 5:** AOM-associated HCRU and costs during pre-PCV13 and post-PCV13 periods, by age groups

	< 18 years			< 5 years			5 to < 18 years		
	Pre-PCV13 (*n* = 12,256)	Post-PCV13 (*n* = 6,629)	*P*-value	Pre-PCV13 (*n* = 7,884)	Post-PCV13 (*n* = 4,370)	*P*-value	Pre-PCV13 (*n* = 4,372)	Post-PCV13 (*n* = 2,259)	*P*-value
**Healthcare resource utilization**									
Inpatient									
Number of visits ^a^, n	12,326	6,657	-	7,935	4,392	-	4,391	2,265	-
Length of hospitalization per visit (days)									
Mean ± SD	4.08 ± 2.89	3.86 ± 3.10	< 0.001*	4.40 ± 2.84	4.15 ± 2.94	< 0.001*	3.50 ± 2.90	3.29 ± 3.32	0.007*
Median (Q1, Q3)	4 (2, 5)	3 (2, 5)	< 0.001*	4 (3, 5)	4 (3, 5)	< 0.001*	3 (2, 4)	3 (2, 4)	< 0.001*
Total length of hospitalization ^b^	50,036	25,579	-	34,725	18,146	-	15,311	7,433	-
Per episode									
Average number of visits	1.01	1.00	-	1.01	1.01	-	1.00	1.00	-
Average length of hospitalization (days)	4.08	3.86	-	4.40	4.15	-	3.50	3.29	-
Outpatient									
Number of visits ^c^, n	11,354	5,806	-	7,375	3,688	-	3,979	2,118	-
Average number of visits per episode	0.93	0.88	-	0.94	0.84	-	0.91	0.94	-
ED									
Number of visits ^d^, n	1,252	688	-	959	545	-	293	143	-
Average number of visits per episode	0.10	0.10	-	0.12	0.13	-	0.07	0.06	-
**Costs**									
Inpatient									
Per visit									
Mean ± SD	17,787.79 ± 17,778.03	21,074.92 ± 34,578.90	< 0.001*	15,617.11 ± 12,569.62	18,524.46 ± 24,757.87	< 0.001*	21,702.18 ± 24,028.49	26,008.74 ± 47,820.97	< 0.001*
Median (Q1, Q3)	13,731.00 (9,653.50, 22,558.00)	16,573.00 (11,760.00, 25,042.00)	< 0.001*	12,710.00 (9,412.50, 18,297.00)	14,915.00 (11,305.00, 21,367.00)	< 0.001*	18,166.00 (10,446.50, 30,092.50)	21,983.00 (13,969.00, 31,352.00)	< 0.001*
Total inpatient costs	218,007,198	139,705,658	-	123,125,266	80,951,908	-	94,881,932	58,753,750	-
Average inpatient costs per episode	17,787.79	21,074.92	-	15,617.11	18,524.46	-	21,702.18	26,008.74	-
Outpatient									
Per visit (excluding pharmacy costs)									
Mean ± SD	840.79 ± 1,374.29	895.08 ± 1,516.02	< 0.001*	860.16 ± 1,414.41	840.34 ± 1,163.90	< 0.001*	805.22 ± 1,296.91	997.90 ± 2,014.17	< 0.001*
Median (Q1, Q3)	455.00 (348.00, 946.00)	475.00 (384.00, 969.00)	< 0.001*	422.50 (368.00, 933.00)	436.00 (422.00, 892.00)	< 0.001*	551.00 (314.00, 960.00)	633.50 (359.00, 1,071.00)	< 0.001*
Total outpatient visit costs	5,750,170	3,276,900	-	3,808,786	2,007,576	-	1,941,384	1,269,324	-
Pharmacy costs per visit									
Mean ± SD	275.96 ± 3,766.34	182.14 ± 191.34	< 0.001*	200.08 ± 250.22	161.88 ± 170.75	< 0.001*	413.39 ± 6,304.55	218.15 ± 218.79	0.168
Median (Q1, Q3)	139 (66, 281)	124 (63, 241)	< 0.001*	130 (59, 268)	114 (58, 211)	< 0.001*	157 (79, 304)	142 (68, 302)	0.044*
Total outpatient pharmacy costs	1,544,544	520,557	-	721,480	296,080	-	823,064	224,477	-
Total outpatient costs ^e^	7,294,714	3,797,457	-	4,530,266	2,303,656	-	2,764,448	1,493,801	-
Per episode									
Average outpatient visit costs	469.17	494.33	-	483.10	459.40	-	444.05	561.90	-
Average pharmacy costs	126.02	78.53	-	91.51	67.75	-	188.26	99.37	-
Average outpatient costs	595.20	572.86	-	574.62	527.15	-	632.31	661.27	-
ED									
Per visit									
Mean ± SD	2,633.93 ± 1,197.78	3,447.39 ± 1,698.16	< 0.001*	2,742.62 ± 1,234.00	3,582.20 ± 1,639.01	< 0.001*	2,285.98 ± 998.75	2,935.68 ± 1,823.22	< 0.001*
Median (Q1, Q3)	2,692.00 (1,853.00, 3,338.00)	3,542.00 (2,229.00, 4,425.00)	< 0.001*	2,834.50 (1,953.00, 3,425.00)	3,746.00 (2,521.00, 4,530.00)	< 0.001*	2,344.00 (1,690.00, 2,843.00)	2,777.00 (1,660.00, 3,681.00)	< 0.001*
Total ED costs	3,242,369	2,347,672	-	2,572,578	1,930,806	-	669,791	416,866	-
Average ED costs per episode	264.55	354.15	-	326.30	441.83	-	153.20	184.54	-
Total costs ^f^	228,544,281	145,850,787	-	130,228,110	85,186,370	-	98,316,171	60,664,417	-
Average total costs per episode	18,647.54	22,001.93	-	16,518.03	19,493.45	-	22,487.69	26,854.55	-

Abbreviations: AOM, acute otitis media; ED, emergency department; HCRU, healthcare resource utilization; PCV, pneumococcal conjugate vaccine; Q1, 25th percentile; Q3, 75th percentile; SD, standard deviation

^a^ Number of inpatient visits is defined as the number of AOM-associated hospital admissions

^b^ Total inpatient hospitalization is defined as the total duration of stay (in days) for all AOM-associated hospitalizations

^c^ Number of outpatient visits is defined as the number of hospital outpatient visits that occur on separate dates. Multiple records on the same date will be considered as a single visit

^d^ Number of ED visits is defined as the number of AOM-associated visits to the hospital ED that occur on separate dates

^e^ Total outpatient costs is defined by the sum of total outpatient visit costs and total outpatient pharmacy costs

^f^ Total costs is defined by the sum of total inpatient costs, total outpatient costs and total ED costs

### Sensitivity analyses

Annual hospitalized IRs for IPD and pneumonia in sensitivity analyses, where non-specific ICD codes were used, showed similar trends to those in the main analyses. ITSA results for quarterly IPD IRs in sensitivity analyses did not show a statistically significant change in trend in the post-PCV13 period (IRR = 1.00, p-value = 0.752), while statistical significance was detected in the main analyses (IRR = 0.58, p-value = 0.001). Statistical significance of ITSA results for quarterly pneumonia IRs in sensitivity analyses were similar to those in the main analyses.

## Discussion

To our knowledge, this is the first study in Taiwan which quantified the clinical and economic burden associated with IPD, non-bacteremic pneumococcal pneumonia, and AOM in children, before and following inclusion of PCV13 into the CIP using the NHIRD.

ITSA results showed that baseline IPD IR in children < 18 years was significantly lower in the post-PCV13 period compared with the pre-PCV13 period, concurring with results from previously reported studies [2–7]. However, we noted a relatively stable IR trend in the post-PCV13 period. This low and stable trend of IR in the post-PCV13 period was likely related to the reduction in susceptible children prior to introduction of PCV13 into the CIP. We observed that the IR of IPD was on a declining trend prior to the addition of PCV13 into Taiwan’s CIP, and remained relatively stable in the post-PCV13 period. One reason for the observed declining trend was the widespread use of PCVs in Taiwan before 2015, due to the introduction of the public-funded national PCV program in 2009 and a PCV13 catch-up program from 2013 to 2014 [20–22]. Thus, the declining trend was not unexpected. In addition, the catch-up program resulted in a high PCV13 coverage of 77.9% among children aged between 1 and 5 years in 2014 [31]. These factors combined contributed to a smaller pool of susceptible children before the inclusion of PCV13 into the CIP, and resulted in the low and stable IR in the post-PCV13 period.

In our study, the trends of non-bacteremic pneumococcal pneumonia and AOM IRs declined in the pre-PCV13 period and increased in the post-PCV13 period. As with IPD, the widespread use of PCVs before the inclusion of PCV13 into the CIP probably led to the declining trends observed in non-bacteremic pneumococcal pneumonia and AOM during the pre-PCV13 period. One possible explanation for the increasing trends of non-bacteremic pneumococcal pneumonia and AOM observed in the post-PCV13 period was the increase in prevalence of non-vaccine serotypes (NVTs) due to selection pressure following universal PCV immunization. This phenomenon is known as serotype replacement. Studies conducted in Taiwan after the introduction of PCV13 have found that NVTs have replaced PCV13 serotypes to become the leading serotypes in Taiwan [22, 32, 33]. It has been suggested that serotype replacement takes place mainly in non-invasive pneumococcal disease among younger children [33]. Hence, the increasing prevalence of NVTs, which may be occurring faster in non-bacteremic pneumococcal pneumonia or AOM, could have resulted in the greater rebound of non-invasive diseases during the post-PCV13 period of our study. However, we were unable to conclusively determine if this was the case in our study since we did not have information on the disease-causing serotypes and episodes in this study were identified based on ICD codes instead of diagnostic tests.

Another possible reason for the increasing trend seen in AOM during the post-PCV13 period was an increase in other pathogens causing AOM. Apart from *S. pneumoniae*, AOM is also commonly caused by non-typeable *Haemophilus influenzae*, *Moraxella catarrhalis* and Group A streptococcus [34]. ICD codes used in this study to identify AOM episodes were not specific to *S. pneumoniae* infections, and it would be difficult to confirm the AOM episodes as due to pneumococcal infections in the absence of laboratory or microbial culture confirmation. A study in a local hospital reported that the proportion of *S. pneumoniae* among samples taken from children < 18 years diagnosed with AOM or chronic otitis media with effusion was on the decline since 2012, while that of non-typeable *Haemophilus influenzae* mirrored the change with an increase from 2012 [35]. Although the period of study was only until 2015, the trends could have continued past 2015, resulting in the increase in AOM during the post-PCV13 period.

In this study, we estimated the HCRU and costs associated with IPD, non-bacteremic pneumococcal pneumonia, and AOM episodes to evaluate the economic burden of pneumococcal diseases. Among the three diseases, HCRU and costs per visit and/or disease episode were generally highest in IPD, followed by non-bacteremic pneumococcal pneumonia and AOM, which was generally also in decreasing order of disease severity. Since total costs were largely driven by inpatient costs, it is expected that total costs will be strongly correlated with disease severity and length of hospitalization. However, total costs were highest for non-bacteremic pneumococcal pneumonia, followed by AOM and IPD. The considerably greater healthcare spending on non-bacteremic pneumococcal pneumonia and AOM was due to the greater number of non-bacteremic pneumococcal pneumonia and AOM episodes, compared to IPD episodes.

### Limitations

This study has several limitations which should be taken into consideration when interpreting the results. ICD codes were used to identify disease episodes, and misclassification of episodes as pneumococcal disease (particularly for AOM) may occur. While the list of pneumococcal-specific ICD codes was developed using literature and in consultation with clinical experts, the accuracy of codes was not validated against laboratory or microbial culture results (which are not available in NHIRD). In addition, inpatient records were used to identify disease episodes. This may underestimate the incidence and healthcare burden of pneumococcal diseases, particularly for AOM. AOM is usually treated in the outpatient settings and patients may only be hospitalized when the condition is severe or when there are complications. Even though non-hospitalized episodes were excluded, hospitalizations are likely the main driver of healthcare burden in Taiwan. Another limitation is that NHIRD is a national claims database, and hence self-pay visits or out-of-pocket expenses are not captured. Thus, the estimated costs associated with pneumococcal diseases in this study may be underestimated. However, as treatment for pneumococcal diseases is covered by the National Health Insurance, we believe that the number of self-paying patients will be small for the diseases studied.

## Conclusion

Residual clinical and economic burden of IPD, non-bacteremic pneumococcal pneumonia, and AOM remained substantial after the inclusion of PCV13 into Taiwan’s CIP. To further reduce the disease burden among children, additional research to investigate the cause of increasing trends of hospitalized non-bacteremic pneumococcal pneumonia and AOM in the post-PCV13 era will be required. Continuous surveillance in Taiwan is important to generate local epidemiological information, detect emerging serotypes, as well as generate knowledge for future vaccine development and policy.

## Electronic supplementary material

Below is the link to the electronic supplementary material.

Supplementary Material 1: Title of data: International Classification of Diseases (ICD), Ninth and Tenth revisions, codes for IPD, pneumonia and AOM. Description of data: ICD codes used to identify hospitalization episodes in this study.

## Data Availability

The datasets generated and/or analyzed during the current study are not publicly available. The data analysed in this study was obtained from the Taiwan’s National Health Insurance Research Database, which is provided by the National Health Insurance Administration and maintained by the Health and Welfare Data Science Center (https://dep.mohw.gov.tw/DOS/cp-5119-59201-113.html), Ministry of Health and Welfare, Executive Yuan, Taiwan. The release of the claims dataset is prohibited by the Taiwanese government. Additional details on International Classification of Diseases codes used in this study are available in Additional File 1.

## Abbreviations

AOM: Acute otitis media
CFR: Case fatality rate
CIP: Childhood Immunization Program
HCRU: Healthcare resource utilization
ICD: International Classification of Diseases
IPD: Invasive pneumococcal disease
IR: Incidence rate
IRR: Incidence rate ratio
ITSA: Interrupted time series analyses
NHIRD: National Health Insurance Research Database
NTD: New Taiwan Dollars
PCV: Pneumococcal conjugate vaccine
PCV13: 13-valent pneumococcal vaccine
