# Supplementary material for: A population-based study on the burden of hospitalized pediatric pneumococcal disease in Taiwan before and after the introduction of 13-valent pneumococcal conjugate vaccine into the childhood immunization program in 2015
Source: BMC Infect Dis. 2025 Feb 5;25:176. doi: 10.1186/s12879-024-10379-z (PMC11800406; doi:10.1186/s12879-024-10379-z)
Supplement: Supplementary file 1 — Supplementary Material 1: Title of data: International Classification of Diseases (ICD), Ninth and Tenth revisions, codes for IPD, pneumonia and AOM. Description of data: ICD codes used to identify hospitalization episodes in this study. [file 12879_2024_10379_MOESM1_ESM.docx]

**International Classification of Diseases (ICD), Ninth and Tenth revisions, codes for IPD, pneumonia and AOM**

| **Disease** | **Category** | **ICD9** | **ICD10** | **Description** |
| --- | --- | --- | --- | --- |
| IPD | Pneumococcal-specific | 038.2;  038.0+041.2;  038.9+041.2;  790.7+041.2;  320.1;  320.2+041.2;  320.8x/320.9 +041.2;  322.9+041.2  510.x+041.2;  511.1/511.9 +041.2;  038.2+ACP code;  038.0+041.2+ACP code;  038.9+041.2+ACP code;  790.7+041.2+ACP code;  038.0+481;  038.9+481;  790.7+481;  041.2;  567.1;  420.9x+041.2;  421.0+041.2;  421.1/421.9 +041.2;  567.23+041.2;  730.0x, 730.2x +041.2;  711.0x/711.9x +041.2 | A40.3;  A40.9+B95.3;  A41.9+B95.3;  R78.81+B95.3;  G00.1;  G00.2+B95.3;  G00.8/G00.9 +B95.3;  G03.9+B95.3;  J86.x+B95.3;  J90/J91.8+B95.3;  A40.3+ACP code;  A40.9+B95.3+ACP code;  A41.9+B95.3+ACP code;  R78.81+B95.3+ACP code;  A40.9+J13;  A41.9+J13;  R78.81+J13;  M00.1x;  K65.8+B95.3;  I30.1+B95.3;  I33.0+B95.3;  I33.9 +B95.3;  K65.2+B95.3;  M86.1x/M86.2x/M86.9 +B95.3;  M00.0x, M00.2x, M00.8x, M00.9+B95.3 | Pneumococcal septicemia  Streptococcal septicemia + PNI  Unspecified septicemia + PNI  Bacteremia + PNI  Pneumococcal meningitis  Streptococcal meningitis + PNI  Bacterial meningitis, unspecified + PNI  Meningitis, unspecified + PNI  Empyema + PNI  Pleural effusion + PNI  Pneumococcal arthritis  Pneumococcal peritonitis  Infective pericarditis + PNI  Bacterial endocarditis + PNI  Endocarditis, unspecified + PNI  Spontaneous bacterial peritonitis + PNI  Osteomyelitis + PNI  Pyogenic/unspecified arthritis + PNI |
|  | Non-specific | 038.0;  038.9;  790.7;  320.2;  320.8x/320.9;  322.9;  510.x;  511.1/511.9;  038.0+ACP code^a^;  038.9+ACP code^a^;  790.7+ACP code^a^  420.9;  421.0;  421.1/421.9;  567.23;  730.0x, 730.2x;  711.0x/711.9x | A40.9;  A41.9;  R78.81;  G00.2;  G00.8/G00.9;  G03.9;  J86.x;  J90/J91.8;  A40.9+ACP code ^a^;  A41.9+ACP code ^a^;  R78.81+ACP code ^a^;  I30.1;  I33.0;  I33.9;  K65.2;  M86.1x/M86.2x/M86.9;  M00.0x, M00.2x, M00.8x, M00.9 | Streptococcal septicemia  Unspecified septicemia  Bacteremia  Streptococcal meningitis  Bacterial meningitis, unspecified  Meningitis, unspecified  Empyema  Pleural effusion  Infective pericarditis  Bacterial endocarditis  Endocarditis, unspecified  Spontaneous bacterial peritonitis  Acute or unspecified osteomyelitis  Pyogenic/unspecified arthritis |
| Non-bacteremic pneumococcal pneumonia | Pneumococcal-specific | 481;    482.9+041.2;  485+041.2;  486+041.2 | J13;  J18.1+B95.3;  J15.x+B95.3;  J18.0+B95.3;  J18.9+B95.3 | Pneumococcal pneumonia  Lobar pneumonia, unspecified + PNI  Bacterial pneumonia, unspecified+ PNI  Bronchopneumonia + PNI  Pneumonia, organism unspecified + PNI |
|  | Non-specific | 482.9;  485;  486 | J15.x/J18.1;  J18.0;  J18.9 | Bacterial pneumonia, unspecified  Bronchopneumonia  Pneumonia, organism unspecified |
|  | All-cause pneumonia (ACP) | 480.x (480.0-480.3, 480.8, 480.9);    481;  482.x;  483.x (483.0, 483.1, 483.8);  484.x (484.1, 484.3, 484.5-484.8);  485;  486;  487.0 | J12.x (J12.0, J12.1, J12.2, J12.3, J12.81, J12.89, J12.9);  J13, J18.1;  A48.1, J14, J15.0, J15.1, J15.2x (J15.20, J15.211, J15.212, J15.29), J15.3, J15.4, J15.5, J15.6, J15.8, J15.9;  J15.7, J16.x (J16.0, J16.8);  A22.1, A37.91, B25.0, B44.0, J17;    J18.0;  J18.2, J18.8, J18.9;  J11.0x (J11.00, J11.08) | Viral pneumonia  Pneumococcal pneumonia  Other bacterial pneumonia  Pneumonia due to other specified organism  Pneumonia in infectious diseases classified elsewhere  Bronchopneumonia, organism unspecified  Pneumonia, organism unspecified  Influenza with pneumonia |
| AOM | AOM | 382.x | H66.xxx;  H67.x | Acute suppurative OM, suppurative and unspecified OM |
|  | OME ^a^ | 381.x;  384.0x | H65.xxx;  H68.xxx;  H69.xx;  H73.0x | Non-suppurative OM and eustachian tube disorders  Acute myringitis, unspecified |

Abbreviations: ACP, all-cause pneumonia; AOM, acute otitis media; ICD, International Classification of Diseases; IPD, invasive pneumococcal disease; OM, otitis media; OME, otitis media with effusion; PNI, pneumococcal infection.

^a^ Excludes 481 (ICD-9) and J13 (ICD-10)
